# Cryo-EM Structure of Isomeric Molluscan Hemocyanin Triggered by Viral Infection

**DOI:** 10.1371/journal.pone.0098766

**Published:** 2014-06-02

**Authors:** Hongtao Zhu, Jun Zhuang, Hongli Feng, Rongfeng Liang, Jiangyong Wang, Lianhui Xie, Ping Zhu

**Affiliations:** 1 National Laboratory of Biomacromolecules, Institute of Biophysics, Chinese Academy of Sciences, Beijing, China; 2 Fujian Provincial Key Laboratory of Plant Virology, Institute of Plant Virology, Fujian Agriculture and Forestry University, Fuzhou, China; 3 Key Laboratory of Biopesticide and Chemical Biology, Fujian Agriculture and Forestry University, Ministry of Education, Fuzhou, China; 4 South China Sea Fisheries Research Institute, Chinese Fisheries Academy, Guangzhou, China; 5 University of the Chinese Academy of Sciences, Beijing, China; The Chinese University of Hong Kong, China

## Abstract

Hemocyanins (Hcs) of arthropods and mollusks function not only as oxygen transporters, but also as phenoloxidases (POs). In invertebrates, PO is an important component in the innate immune cascade, where it functions as the initiator of melanin synthesis, a pigment involved in encapsulating and killing of pathogenic microbes. Although structures of Hc from several species of invertebrates have been reported, the structural basis for how PO activity is triggered by structural changes of Hc *in vivo* remains poorly understood. Here, we report a 6.8 Å cryo-electron microscopy (cryo-EM) structure of the isomeric form of hemocyanin, which was isolated from Abalone Shriveling syndrome-associated Virus (AbSV) infected abalone (*Halitotis diversicolor*), and build a pseudoatomic model of isomeric *H. diversicolor hemocyanin 1* (HdH1). Our results show that, compared with native form of HdH1, the architecture of isomeric HdH1 turns into a more relaxed form. The interactions between certain functional units (FUs) present in the native form of Hc either decreased or were totally abolished in the isomeric form of Hc. As a result of that, native state Hc switches to its isomeric form, enabling it to play its role in innate immune responses against invading pathogens.

## Introduction

Hemocyanins (Hcs) are blue respiratory macromolecules that contain copper ions, a characteristic that enables Hcs to function as oxygen transporters in a variety of arthropods and mollusks [1]. Some Hcs, such as the *keyhole limpet hemocyanin 1* (KLH1), possess a great amount of epitopes which can efficiently trigger an immune response, and therefore have been widely used as antigen carrier proteins or non-specific immune stimulation (NSI) proteins [2]–[5].

Hcs from different organisms display diversified structures. In arthropods, for instance, Hcs exist as hexamers as well as multihexamers, whereas in molluscan, Hcs were shown to display deca-, dideca-, as well as tridecameric forms [1], [2], [5]. As the oxygen carrier and transporter in *Halitotis diversicolor*, each functional unit (FU) of *H. diversicolor* Hemocyanin contains an oxygen-binding center, which contains two copper ions directly ligated to the side chains of three conserved histidines surrounding the copper ions [6], [7]. Upon contact with oxygen, the Cu ion pair in each FU binds to one peroxide ion, turning the Cu(I)-Cu(I) deoxygenated state into an oxidated Cu(II)-Cu(II) state [8], [9], after which the colorless Hc turns blue.

Following invasion of mollusks or arthropods by pathogens such as viruses, Hcs become involved in the launch of an innate immune response through activation of the innate phenoloxidase (PO) propensity [10]–[13]. Interestingly, the oxygen-binding centre of Hc also serves as the active site of the PO entity [14], [15]. It was reported earlier that after undergoing a series of conformational changes *in vitro*, native-state Hc (i.e. inactive PO) can switch from being an oxygen carrier to catalytically active PO. However, this functional switch only occurred after specific stimuli such as lipopeptides, detergents and proteinase were added *in vitro* [16]–[20]. Recent *in vivo* studies in molluscs suggested that the inactive pro-phenoloxidase (proPO) conformation will be turned into an active PO conformation by specific recognition proteins [21], [22]. This elaborate activation system controls the proPO activation and the innate immune response in mollusks [1].

Although the structure of native form molluscan Hc had been reported [2], [23], the structure basis how molluscan Hc switches its role from mainly acting as an oxygen carrier to becoming a PO remains poorly understood. In this study, we found that isomeric Hcs from abalone infected with abalone shriveling syndrome-associated virus (AbSV) [24] exhibit strong PO activity in the absence of *in vitro* allosteric effectors. The architecture of isomeric form of Hc from AbSV infected abalone turns into a more relaxed state compared with native form of Hc. Our results suggest that the decrease in the interactions between FUs in isomeric Hc contributes to the activation of PO activity of HC. As a result, the Hc switches its role from being mainly an oxygen carrier to functioning in its capacity as an active PO, required for the proper initiation of a mollusk innate immune response.

## Results

### The overall structure of isomeric HdH1 induced by AbSV infection

**Figure 1:**
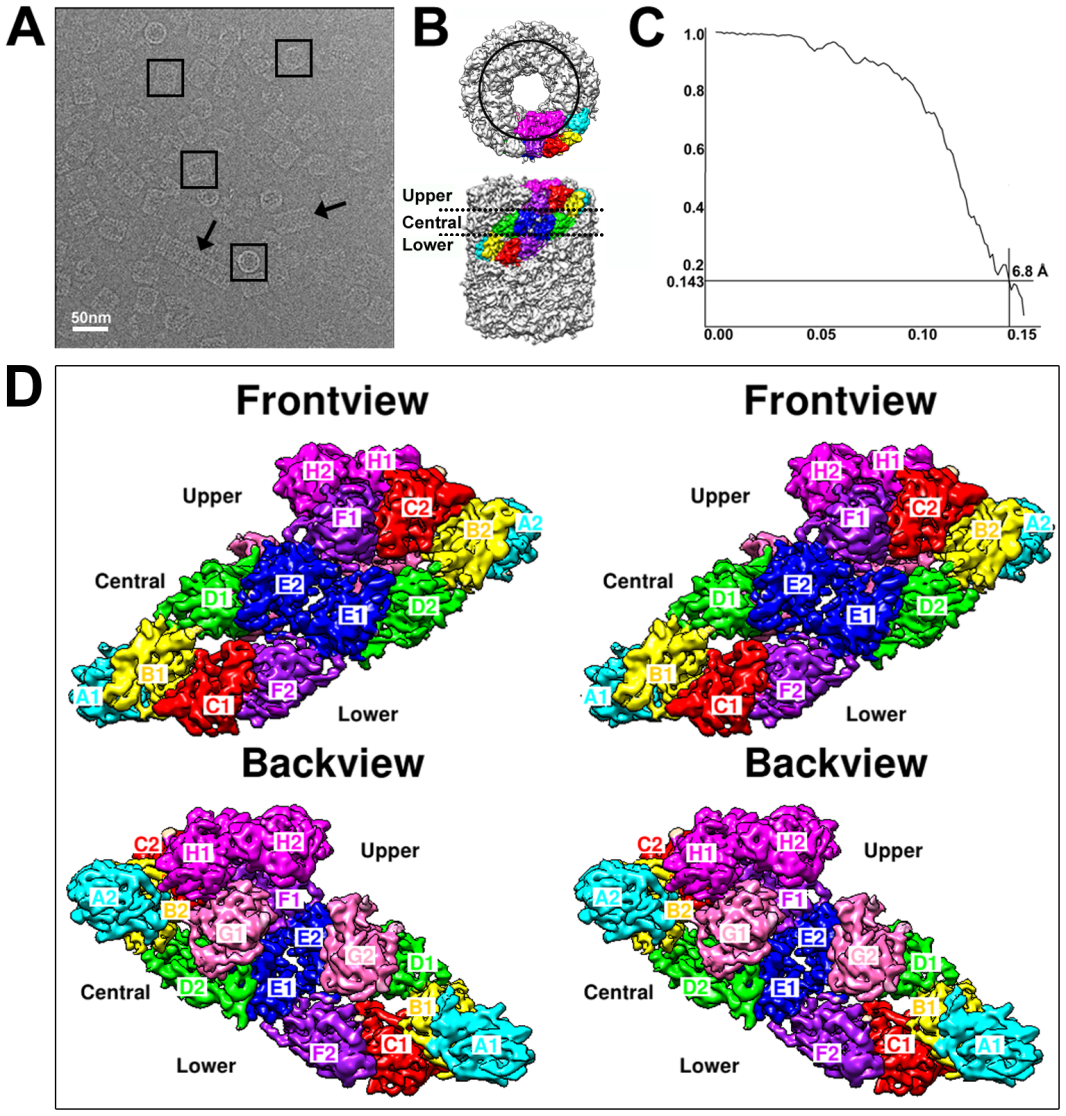
The overall structure of isomeric HdH1. (A) A representative cryo-EM micrograph of isomeric HdH1. The bacilliform Hc-like particles are indicated by black arrows and the didecameric particles used in the 3D reconstruction are marked with black boxes. (B) Top view and side view of 3D reconstructed map of isomeric HdH1. An asymmetric unit is highlighted and its component Function Units (FUs) are marked with different colors. The slab area containing FU_H1 and H2 is circled by a black line in the top view panel. The upper, central and lower tiers are labeled. (C) FSC curve of isomeric HdH1 reconstruction according “gold-standard” criterion [26], [27]. (D) Stereo pair views of FUs in one asymmetric unit of isomeric HdH1. The FUs are named as A1 through H1, A2 through H2 and are indicated in different colors. Backview is with a rotation of 180 degree from the front view.

Hcs in the AbSV infected abalone were purified, cryo-EM imaged and subjected to 2D analysis and 3D reconstruction by single particle analysis. Similar to our previous observation [24], a part of isomeric HdH1 particles from viral infected abalone showed as rod-shaped Hc-like particles, i.e., tridecamers or multidecamers (indicated by arrows in Fig. 1A), which was also reported in the EM observation of *keyhole limpet hemocyanin 2* (KLH2) decamer when dialyzing against a high calcium and magnesium concentration [25]. Only the didecameric Hc particles of isomeric HdH1 were selected (black boxes in Fig. 1A) for further analysis and 3D reconstruction. The reconstructed isomeric form of HdH1 from AbSV infected abalone exhibits a D5 symmetry and encompasses two cylindrical decamers (Fig. 1B), reminiscent to the structure of native form of HdH1 recently resolved by Zhang *et al* at 4.5 Å resolution [23] and to the structure of native KLH1 previously resolved by Gatsogiannis *et al* at 9 Å resolution [2]. Each decamer is composed of five asymmetric units containing 8 pairs of homologous FUs that are inversely arranged. Thus, a total of 160 FUs form a hollow cylindrical didecameric particle in HdH1 (Fig. 1B, movie S1). Our structure of the isomeric form of HdH1 was reconstructed at 6.8 Å resolution, determined by gold-standard Fourier Shell Correlation (FSC) criterion at the 0.143 cutoff [26], [27] (Fig. 1C), using 57,043 particles selected from 2379 cryo-EM micrographs. The reconstruction results show that the decamer of the isomeric HdH1 consists of three tiers: A1-B1-C1-F2 in the lower tier, D1-E2-E1-D2 in the central tier with G2-G1 attached inside, and A2-B2-C2-F1 in the upper tier together with the slab-forming H1-H2 (Fig. 1D, movie S1). Overall, the relative locations and the arrangement of the FUs in the isomeric form of HdH1 are nearly identical to those in the native form of HdH1 [23], with a correlation score of 0.95 at 6.8 Å resolution, and to those of KLH1 [2] as well, except certain minor differences between corresponding FUs (movie S2) and a few density spikes located in the central tier in the FU-Es of isomeric HdH1 (Fig. S1), which may be attributed into the glycosylations similar to those in KLH1 as suggested by Gatsogiannis *et al* [2].

### The pseudoatomic models of FUs of isomeric HdH1

Each of the FUs of HdH1 exhibits a high degree of sequence identity to FU_G of *Octopus dofleini* Hc (Odg) and FU_E of *Rapana thomasiana* Hc (RtH2e) (Fig. S2), whose structures had been solved previously by x-ray crystallography at an atomic resolution [6], [7]. The high degree of conservation in the protein sequences of FUs of Hc from various species allows us to build pseudoatomic models of the isomeric HdH1 FUs by combining the cryo-EM 3D reconstruction and homologue modeling (Fig. S3, and as described in **Materials and Methods)**. Similar to the structures of FUs in native HdH1 (PDB ID: 3J32) [23], all FUs of isomeric HdH1 contain two structural domains, the N-terminal core domain, which predominantly consists of α-helices, and the C-terminalβsheet domain, which contains theβsheets region (dashed lines in Fig. S3). FU_H, which locates in the end and forms the slab of cylindrical decamers (Fig. 1B and 1D, magenta domains), is structurally unique among all of the FUs of HdH1 as it has an additional cupredoxin-like fold (Fig S2, sequence underlined and Fig. S3, red densities) [28], [29]. All of the built pseudoatomic models of isomeric FUs fit well with the corresponding density maps of isomeric HdH1, while the α helices,βsheets and the loops of each FU can be accommodated precisely in the density maps (Fig. S3).

### The isomeric HdH1 from AbSV-infected display strong PO activity

The main function of molluscan Hc is to transport oxygen, similar to hemoglobin in vertebrates. However, in response to invading external pathogens, Hc is able to switch its activity from binding oxygen to oxidizing phenol. Phenoloxidases (POs) play an important role in the innate immune responses of invertebrates. Activated POs in invertebrates are used to initiate the synthesis of brown melanin, which subsequently encapsulates and inactivate the pathogens [10], [30], [31]. The activation of PO usually comprises two steps, i.e., the formation of o-diphenols from the hydroxylation of monophenols catalyzed by tyrosinases (Ty), and the subsequent oxidation of diphenols into quinones converted by catecholoxidases (CO) and tyrosinases [8], [31].

**Figure 2:**
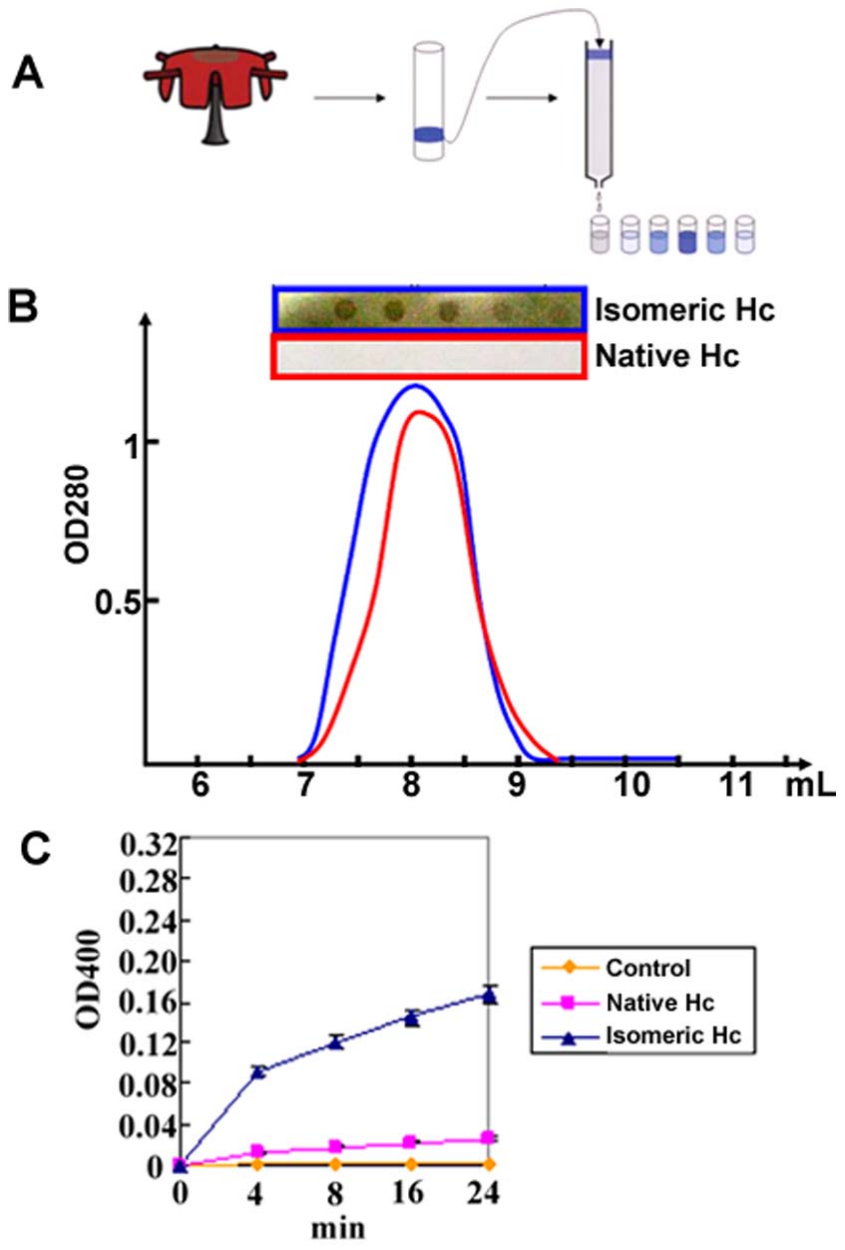
The purification profile of Hcs and phenoloxidase assay from native and isomeric Hcs. (A) Flow chart for Hcs purification. (B) Eluted fractions of isomeric (blue) and native Hc (red) were tested for phenoloxidase activity by OD 280 and dot-blot experiments using 1 mM o-diphenol. (C) Spectroscopic analysis of phenoloxidase activity from isomeric (blue) and native (pink) Hcs. The control (orange) is the same buffer as used to dissolve Hcs.

We previously reported that the AbSV-infected abalones were characterized with melanization of pleopods, which suggests a symptom closely related to the activation of Hc-derived PO in abalone [24]. To explore whether the AbSV infection induce the PO activity of the Hcs in abalone, we analyzed the CO activities of Hc isolated from both healthy and AbSV-infected abalones and found that the isomeric Hc from AbSV-infected abalone exhibited significantly stronger CO activity than native Hc at both qualitative and semi-quantitative levels (Fig. 2). These results suggested that Hcs were effectively converted from respiratory proteins to functional POs following AbSV infection in vivo, reminiscent of the activation of Hc-derived PO by artificial inducers [10], [32].

### Decrease in interaction between FUs of isomeric HdH1

**Figure 3:**
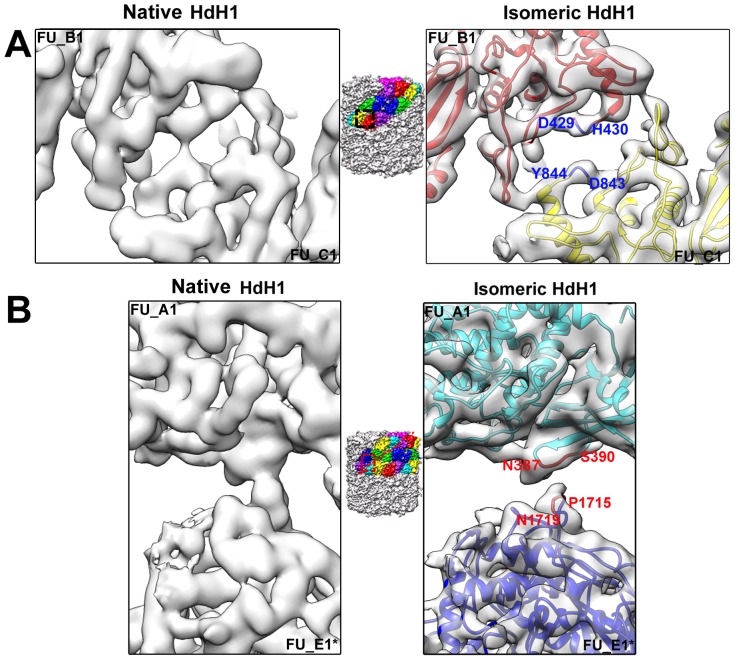
Decrease of FU-FU interactions in the cryo-EM density maps of isomeric HdH1. Left panels: density maps of native HdH1 which are low-pass filterer to 6.8 Å from the reported 4.5 Å cryo-EM structure [23]. Right panels: density maps of isomeric HdH1 reconstructed at 6.8 Å resolution in this study, filled with the corresponding pseudo-atomic models as shown in Fig. S3. (A) An example of loss of interaction between FUs inside one asymmetric unit, i.e., FU_B1 (red) and FU_C1 (yellow). The location of this FU-FU interaction in the didecamers is highlighted by a black box in the middle insert. Residues possibly involved in the interaction are labeled in the right panel. (B) An example of interaction loss between FUs from neighboring asymmetric units, i.e., FU_A1 (cyan) and FU_E1* (blue). A dot red line is used to indicate the boundary of two asymmetric units. The location of the interaction is highlighted by a red box in the middle insert. Residues possibly involved in the interaction are labeled in the right panel.

**Figure 4:**
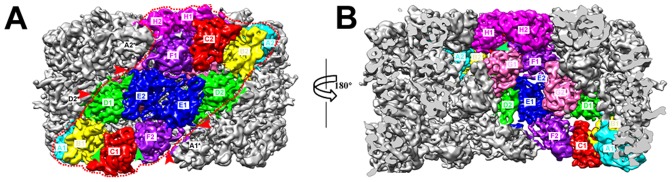
The distribution of decreased FU-FU interactions in isomeric HdH1. The decreased FU-FU interactions inside one asymmetric unit are indicated with green arrows, while the decreased FU-FU interactions between asymmetric units are indicated with red arrows. (A) The side view of isomeric HdH1. The boundaries of one asymmetric unit is marked with red dashed lines. All FUs in the asymmetric unit are colored as Fig. 1. (B) Longitudinal section view of the isomeric HdH1 with a rotation of 180 degree from (A).

To explore the structural changes of molluscan Hc in vivo introduced by viral infection, we low-pass filtered the reported 4.5 Å cryo-EM density map of native HdH1 [23] to 6.8 Å, the same resolution as our reconstructed cryo-EM density map of isomeric HdH1 isolated from AbSV-infected abalone, and compared these two density maps in all of the FU regions. While the overall shape of the isomeric HdH1 appears similar to the native HdH1 (Fig. S1), the interactions between neighboring FUs of isomeric HdH1 become weaker or looser (Fig. 3, Fig. S4, Fig. S5A and Fig. 4). A subset of the FU-FU interactions, either inside the same asymmetric unit (i.e., B1-C1, C1-F2, H1-G1) (Fig. 3A, Fig. S4, green arrows in Fig. 4), or from neighboring asymmetric units (i.e., A1-E1*, A2*-E2, D1-D2*, A1*-F2, where ‘*’ indicates the FU from another asymmetric unit) (Fig. 3B, Fig. S5A, red arrows in Fig. 4), become either weaker or were found to be completely abolished.

### The structure of native HdH1 from healthy abalone

To further explore the differences between native HdH1 and isomeric HdH1, we extracted native Hcs from healthy abalone following the same procedure as for the isomeric Hcs from AbSV infected abalone, and performed the similar cryo-EM analysis and 3-D reconstruction (Fig. S6A-B). Similarly determined by the “gold standard” FSC criterion, the structure of native HdH1 isolated from healthy abalones was reconstructed at about 8.4 Å resolution (Fig. S6C). Although at a relatively lower resolution, the overall architecture of native HdH1 in this study is almost identical to the reported structure of native HdH1 by Zhang *et al* [23] at a higher resolution (Fig. S6B, D), with a correlation score of 0.96 at the 8.4 Å resolution level. Interestingly, a subtly low correlation score 0.95 was calculated between our native HdH1 map and the isomeric HdH1 map, which was similarly low-pass filtered to 8.4 Å for correlation calculation. Moreover, it is worth noting that the previously indicated FU-FU interactions loss (either inside one asymmetric unit or between asymmetric units) in the isomeric HdH1 (Fig. 3, Fig. 4, Fig. S4 and Fig. S5) as compared with the density map of native HdH1 reconstructed by Zhang *et al* [23] were found mostly intact in our reconstructed native HdH1 density map (Fig. S6D and unshown data), which suggests that the observed structural difference between isomeric HdH1 and native HdH1 are likely attributed to the AbSV infection other than the reconstruction process. Since the density map of native HdH1 by Zhang *et al* [23] is in a higher resolution relative to that in our reconstruction, it was chosen to be compared with the map of isomeric HdH1 induced by AbSV infection.

## Discussion

### Oxygen-binding and PO activities of isomeric HdH1

It was reported previously that Hc-derived PO can be activated *in vitro* by both detergent such as SDS, as well as phospholipids [20], [33]. However, how exactly Hc-derived PO is activated *in vivo* remains largely unknown.

Hemocyanins (Hcs) of mollusks function not only as oxygen transporters, but also as phenoloxidases (POs). It was reported earlier that only oligomers in molluscan multimeric Hc exhibit cooperative oxygen-binding activity, but not disassembled subunits [34]. The recently determined cryo-EM structure of native HdH1 suggests that the FUs with the oxygen-binding capability will form so-called “communication clusters”, while the interactions between asymmetric units designated as A1-E1*, B1-D2*, A2*-E2, B2*-D1 were reported responsible for the cooperative effects of the oxygen-binding and releasing in native HdH1 [23]. In the structure of isomeric HdH1, we found that the interface communications of A1*-E1 and A2*-E2 were lost (Fig. 3B, Fig. S5A), while the communications between B1-D2* and B2*-D1 remained almost intact, even though the connections appear weaker (Fig. S5B). This may suggest that only part of the oxygen-binding FUs are converted into entities with PO activity, while others may maintain their ability to transport oxygen, which is essential for maintaining the native physiological function of Hc. It is interesting that both forms of interface communications, i.e., oxygen-binding form (B1-D2* and B2*-D1) and potentially PO form (A1*-E1 and A2*-E2), discretely coexist in a single symmetrically cylindrical Hc (Fig. 4). That is, the allosteric Hc may play two roles, i.e., respiratory protein and innate immunity molecule, simultaneously in the AbSV infected abalone to act as the phenoloxidases (POs) while remaining the oxygen binding state. It remains to be seen whether the observed switch between these two roles is based on a reversible as well as stochastic process.

### The formation of long rod-shaped particles in isomeric HdH1

As we previously reported [24], a part of isomeric HdH1 particles from AbSV infected abalone displayed as long rod-shaped Hc-like particles (arrows in Fig. 1A). A similar observation, i.e., long fibers, had also been reported for KLH2 decamer when dialyzing against a high calcium and magnesium concentration *in vitro* [25]. To evaluate whether these observed long bacilliform particles in isomeric HdH1 were induced by the *in vitro* purification conditions used in our study, we simultaneously treated Hc preparations from both healthy and AbSV-infected abalone with identical buffer and procedures (see **Materials and Methods**), and no noticeable long bacilliform particles were found in native form of Hc (Fig. S6A). Thus, the observed long bacilliform morphology of isomeric HdH1, which is likely to be the aggregated form of decamers, was presumably triggered by AbSV infection.

In contrast to a single band with large molecular weight observed in native form of Hc (Fig. S7A), the SDS-PAGE profile of isomeric form of Hc composed of a range of bands with different molecular weights (Fig. S7B). One strong protein band with a molecular weight of 60 kD present only in the isomeric HdH1 sample (black arrow in Fig. S7B) was subsequently identified by tandem mass spectrometry (MS) to be FU_H (Fig. S7C), the FU that forms the slab on top of the cylindrical Hc (Fig. 1B and 1D, magenta color). In agreement with this result, the connection between FU_H1 and its only interaction partner, FU_G1, was found to have decreased in the density map of isomeric HdH1 (Fig. S4B, Fig. 4B). These results suggest that the interfacial connection between FU_G1 and FU_H1 was decreased or lost in isomeric HdH1, possibly induced by AbSV infection, increasing the chance that the so-called “slab” (i.e. FU_H) of isomeric HdH1 dissociates from the cylindrical particle (Fig. 1B, 1D, Fig. S7). As a consequence, the cylindrical architecture of isomeric HdH1 could lengthen into the long bacilliform particle form due to the absence of the “slab” (arrows in Fig. 1A).

### The crosstalk between AbSV infection and Hc-derived PO activation involved in innate immunity

When mollusks are attacked by viruses or other pathogens, e.g., AbSV, certain microbial products such as peptidoglycans or lipopolysaccharides from bacteria will trigger PO activation after recruiting specific recognition proteins, and the stringent regulation (i.e., pathogen-associated molecular pattern, PAMP) will control the extracellular activation [10], [30]. Thus, the innate immune response could be activated in AbSV-infected abalone (*H. diversicolor*) [24].

**Figure 5:**
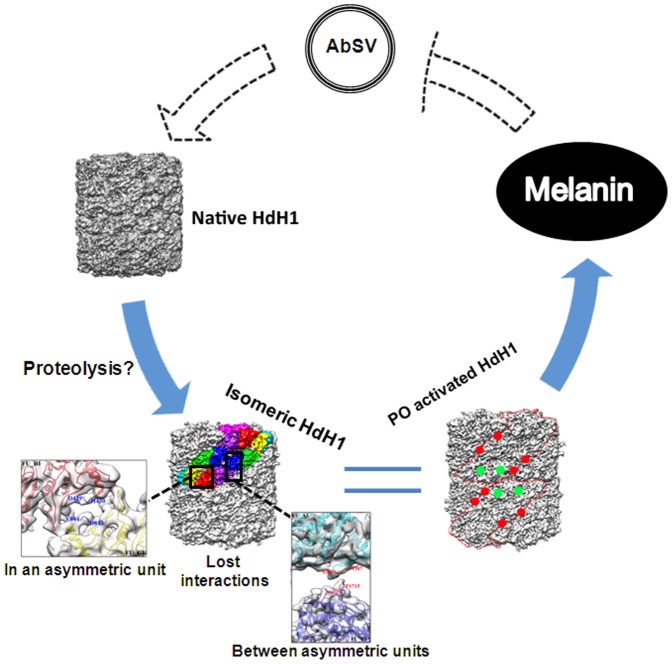
A hypothetical diagram of PO activation in isomeric molluscan hemocyanin triggered by AbSV infection. The red and green dots represent the FU-FU interactions indicated by red and green arrows in Fig. 4, respectively.

Although the precise mechanisms linking PO activity and innate immune response in mollusks remain to be elucidated, our study provides structural insights into what role structural changes of host proteins might play. As illustrated in Fig. 5, upon the attack of AbSV, limited proteolysis may happen in the Hc of AbSV-infected abalone, which elicits the relaxation of FU-FU interactions. While the interaction decrease between FU_G1 and FU_H1 may result in the dissociation of “slab” from the isomeric HdH1, the decreases in other FU-FU interactions, either within one asymmetric unit or between asymmetric units of HdH1, would contribute to the conversion of the native form of Hc into a relaxed isomeric form (i.e., with FU-FU interaction decreased) of Hc and subsequently to the activation of PO. The decrease of interactions among FUs inside one asymmetric unit may contribute to the instability of the asymmetric unit, which increases susceptibility to lose contact with the neighboring FU. The weakening of interaction between neighboring FUs of different asymmetric units is likely to greatly decrease the stability of the overall architecture of isomeric HdH1, possibly due to this interaction defect. The loss or decrease in interaction between FUs in isomeric HdH1 may render the movement of βsheet domains of FUs away from their PO core domains easier, which had been showed that PO activation is mostly associated with [1], [35]. This movement can result in greater exposure of the active PO sites, which ultimately contributes to switching the function of HdH1 to predominantly PO enzymatic activity. After PO activation, isomeric Hc becomes involved in the production of brown melanin, which is then utilized to act against pathogenic microbes such as AbSV, during the innate immune response in mollusks (Fig. 5).

An in-depth understanding of the relationship and the arms race between the innate immune activity of Hc and the counter reaction of viruses and bacteria will help to shed more light on the general theme of co-evolution between pathogens and hosts, and specifically on the evolution of innate immune responses observed as early as in arthropods and mollusks.

## Materials and Methods

### The purification and phenoloxidase identification of Hemocyanins

Hemocyte was harvested from the pleopod of healthy and AbSV-infected abalone, respectively. The hemocyte extract was centrifuged 10,000 g for 10 min at 4 degree. The rude extract of hemocyanin in hemocyte contains mostly highly concentrated hemocyanin, and was used for cryo-EM analysis.

For measurement of phenoloxidase activity, Hcs were further purified through column chromatography using a superdex 200 10/300 column. Protein concentration was determined spectrophotometrically from the absorption at 280 nm. Hemocyanin (Hc) was sedimented in a Optima L-80 XP ultracentrifuge (Beckman Coulter), equipped with a SW 32 rotor, at a speed of 28000 rpm for 18 h through CsCl gradient density at 4°C. The blue band of hemocyanin was absorbed through the syringe along a long needle and subjected to a second ultracentrifugation step at a speed of 40000 rpm for 1.5 h with a Type 45 rotor. The pelleted Hc was suspended using 20 mM phosphate buffer, pH 7.8, containing 2.5 mM MgCl_2_.The o-diphenol oxidase activity of Hcs was assayed at 30°C as previously described [19]. Briefly, different fractions of native Hc at the concentration from 1.0×10^−6^ to 4.0×10^−6^ M, or isomeric Hc at the concentration from 1.5×10^−6^ to 3.4×10^−6^ M were loaded on Hybond N+ membrane. The sampled membrane was then placed into the reaction solution containing 1 mM o-diphenol to initiate the dot reaction. Both native and isomeric Hcs with the same concentration (1.5×10^−6^ M) were subjected to the PO assay through colorimetric analysis and a spectrophotometer was used to monitor the production of quinone at the absorbance of 400 nm.

### Electron Cryomicroscopy

Hcs were diluted to 0.5 mg/ml with 20 mM phosphate buffer, pH 7.4, containing 150 mM NaCl before EM sample preparation. An aliquot of 3.5 µL of the diluted samples was applied to a Quantifoil R2/2 200 mesh holey grid (Quantifoil Micro Tools GmbH, Jena, Germany) and blotted for 4 s in a chamber at 100% humidity using an FEI Vitrobot Mark IV. Images of hemocyanins were taken using an FEI 300-kV Titan Krios cryo-electron microscope equipped with a Gatan UltraScan4000 (model 895) 16-megapixel CCD, operated at accelerating voltage of 300 kV with magnification set to 75,000×,which corresponds to a pixel size of 1.196 Å. The dose for each micrograph was approximately 18 e/Å^2^.

### Image Processing, 3D Reconstruction and Structure Analysis

EMAN2 [36] was initially used for particle picking, CTF correction, 2D classification, projection refinement and 3D reconstruction for isomeric hemocyanin with C5 symmetry imposed. 57,043 isomeric hemocyanin particles were then imported to Relion-1.1 [37] for further refinement and reconstruction by applying D5 symmetry, using the reconstructed map from EMAN2 as the initial model. The final resolution of isomeric HdH1 is measured by Relion-1.1 using a ‘gold-standard FSC’ criterion at 0.143 cutoff, which divided the datasets into two independent parts from the beginning [26], [27]. The reconstructed map was b-factor corrected using Xmipp-2.4 [38] to generate the final density map of isomeric hemocyanin. UCSF Chimera [39] was used for the visualization and the segmentation of different FUs from the final EM density map. The reconstruction of native Hcs was similarly performed with 16,840 particles selected and processed.

The sequences of Hc FUs from different species were aligned in Clustal Omega [40], [41] and further edited in ESPript 2.2 [42], [43]. Structures of each FU in isomeric HdH1 were initially predicted by homologue modeling based on their sequences using the online sever I-TASSER [44], and then flexibly fitted into the corresponding segmented FU density maps of isomeric HdH1 using MDFF [45] to build the pseudoatomic models of isomeric HdH1 FUs.

### Accession numbers

The electron density map of isomeric HdH1 has been deposited in the EMDB under accession code EMD-2503.

## Supporting Information

Figure S1 **Structure comparison between isomeric form and native form of** ***H. diversicolor Hemocyanin 1*** **(HdH1).** The spikes found in isomeric HdH1 are indicated with red arrows. Up panels: the side view of the whole HdH1 maps. Bottom panels: the top view of sections crossing the FU_E in the central tier of HdH1 (dashed lines in the top panels). (A) The density map of native form of HdH1 filtered to 6.8 Å from [23]. (B) The density map of isomeric HdH1 reconstructed at 6.8 Å resolution. (C) Superimpose of isomeric HdH1 map (blue) into the native form of HdH1 (white).
(TIF)

Figure S2 **Sequence alignment of FU_E of** ***Rapana thomasiana*** **Hc (RtH2e), FU_G of** ***Octopus dofleini*** **Hc (Odg) and eight FUs of HdH1.** Six conservative histidines located in the active PO site of each FU are marked with red stars. The sequence of cupredoxin-like domain on FU_H is underlined by a black line.
(TIF)

Figure S3 **Pseudoatomic models of FUs of isomeric HdH1 within an asymmetric unit.** Each FU consists of two domains: the core domain (red dotted line) and the β sheet domain (blue dotted line). The location of the PO active center is indicated by a red star, as shown in FU_A1. The cupredoxin-like domain in FU-H is highlighted in red.
(TIF)

Figure S4 **Decrease in FU-FU interaction inside one asymmetric unit of HdH1.** Left panels: density maps of native HdH1 filtered to 6.8 Å from [23]; Right panels: density maps of isomeric HdH1 filled with the corresponding pseudoatomic models from Fig. S3. The locations of the interactions in the didecamer are indicated by black squares in the inserts. Residues possibly involved in the interactions are labeled in the right panels. (A) Decrease in the interaction between FU_F2 and FU_C1. (B) Decrease in the interaction between FU_G1 and FU_H1.
(TIF)

Figure S5 **Decrease in FU-FU interaction between asymmetric units of HdH1.** Left panels: density maps of normal HdH1 filtered to 6.8 Å from [23]; Right panels: density maps of isomeric HdH1 filled with the corresponding pseudoatomic models from Fig. S3. A red dot line is used to illustrate the boundary of two asymmetric units. The locations of the interactions are indicated by red squares in the inserts. The residues possibly involved in the interactions are labeled in the right panels. (A) Loss of FU-FU interaction between asymmetric units, i.e., FU_A1* and FU_F2, FU_A2* and FU_E2, and FU_D1 and FU_D2*, where * stands for FU from another asymmetric unit. (B) Two FU-FU interactions involved in the oxygen-binding “communication cluster” [1], i.e. FU_D2* and FU_B1, FU_D1 and FU_B2*, are retained in the isomeric HdH1 structure.
(TIF)

Figure S6 **The overall structure of native HdH1 isolated from healthy abalones.** (A) A representative cryo-EM micrograph of native HdH1. The didecameric particles used in the 3D reconstruction are marked with black boxes. (B) Top view and side view of 3D reconstructed map of native HdH1. An asymmetric unit is highlighted and its component Function Units (FUs) are marked with different colors. The slab area containing FU_H1 and FU_H2 is circled by a black line in the top view panel. The top, middle and bottom tiers are labeled. (C) FSC curve of native HdH1 reconstruction according to “gold-standard” criterion. (D) Comparison of cryo-EM density map of isomeric HdH1 isolated from AbSV-infected abalone (middle) with that of native HdH1 reconstructed by Zhang et al [23] (left, low pass filtered to 6.8 Å) and that of native HdH1 isolated from healthy abalone (right). Upper panel: An example of previously indicated FU-FU interaction loss between FUs from neighboring asymmetric units, i.e., FU_A1 (cyan) and FU_E1* (blue), in isomeric HdH1 (middle) as compared with the native HdH1 by Zhang et al [23] was found almost intact in the native HdH1 isolated from healthy abalone (right). A dot red line is used to indicate the boundary of two asymmetric units. The location of the interaction is highlighted by a red box in the small insert panel. Residues possibly involved in the interaction are labeled. Lower panel: An example of FU-FU loss of interaction between FUs inside one asymmetric unit, i.e., FU_B1 (red) and FU_C1 (yellow), is similarly illustrated. The FU-FU interaction lost in the isomeric HdH1 (middle) was found mostly intact in the native HdH1 from healthy abalone. The location of this FU-FU interaction in the didecamers is highlighted by a black box in the insert small panel. Residues possibly involved in the interaction are labeled.
(TIF)

Figure S7 **SDS-PAGE and mass spectrometry (MS) analysis of Hcs. (A) SDS-PAGE analysis of native Hcs.** The lanes 1–3 were loaded with the eluted fractions 8.0, 8.2 and 8.4 of native Hcs in Fig. 2B (red) respectively. (B) SDS-PAGE analysis of isomeric Hcs. The lanes 1–3 were loaded with the eluted fractions 8.0, 8.3 and 8.6 of isomeric Hcs in Fig. 2B (blue) respectively. A protein band around 60 kD was indicated with a black arrow. (C) MS analysis result of the indicated 60 kD protein band in **B**, which corresponds to FU_H of HdH1.
(TIF)

Movie S1 **The overall architecture of isomeric HdH1 isolated from AbSV infected abalone and one asymmetric unit with colored FUs are displayed.**
(MOV)

Movie S2 **The morph video to show changes of a representative FU, FU_B1, between the native and isomeric forms of HdH1.**
(MOV)
