# Supplementary figures and images for: Cryo-EM Structure of Isomeric Molluscan Hemocyanin Triggered by Viral Infection
Source: PLoS One. 2014 Jun 2;9(6):e98766. doi: 10.1371/journal.pone.0098766 (PMC4041863; doi:10.1371/journal.pone.0098766)

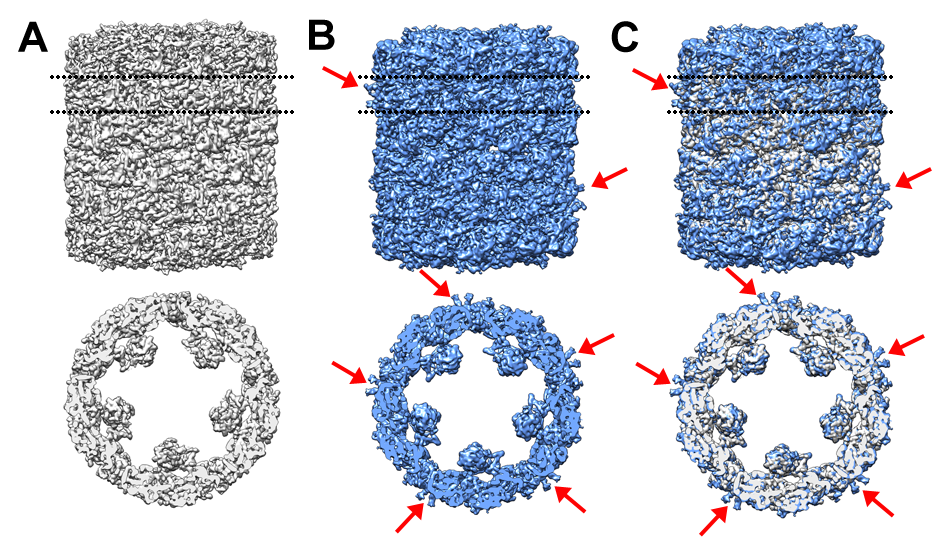

Supplement: Figure S1 — Structure comparison between isomeric form and native form of H. diversicolor Hemocyanin 1 (HdH1). The spikes found in isomeric HdH1 are indicated with red arrows. Up panels: the side view of the whole HdH1 maps. Bottom panels: the top view of sections crossing the FU_E in the central tier of HdH1 (dashed lines in the top panels). (A) The density map of native form of HdH1 filtered to 6.8 Å from [23]. (B) The density map of isomeric HdH1 reconstructed at 6.8 Å resolution. (C) Superimpose of isomeric HdH1 map (blue) into the native form of HdH1 (white). (TIF) [file pone.0098766.s001.tif]

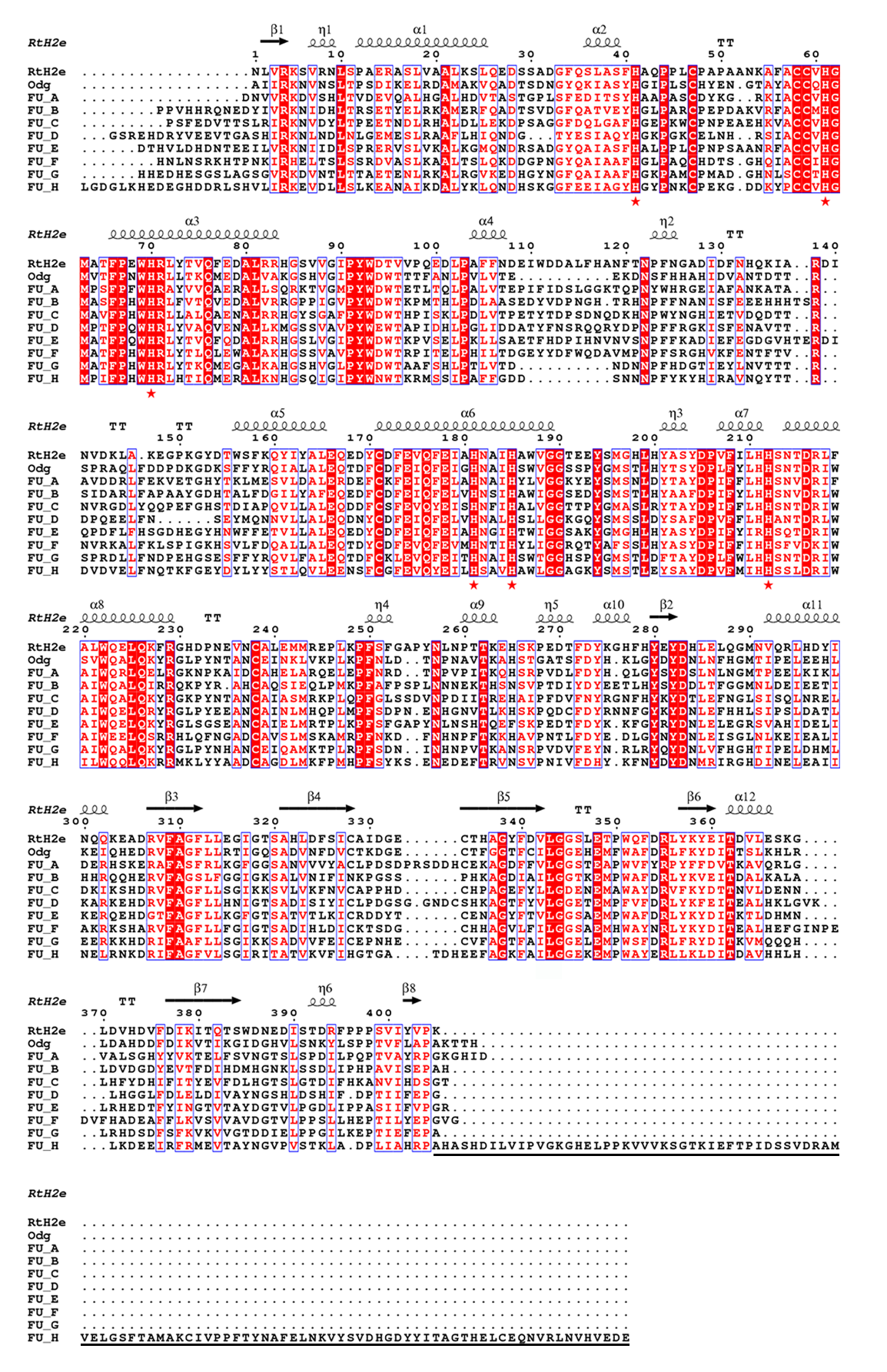

Supplement: Figure S2 — Sequence alignment of FU_E of Rapana thomasiana Hc (RtH2e), FU_G of Octopus dofleini Hc (Odg) and eight FUs of HdH1. Six conservative histidines located in the active PO site of each FU are marked with red stars. The sequence of cupredoxin-like domain on FU_H is underlined by a black line. (TIF) [file pone.0098766.s002.tif]

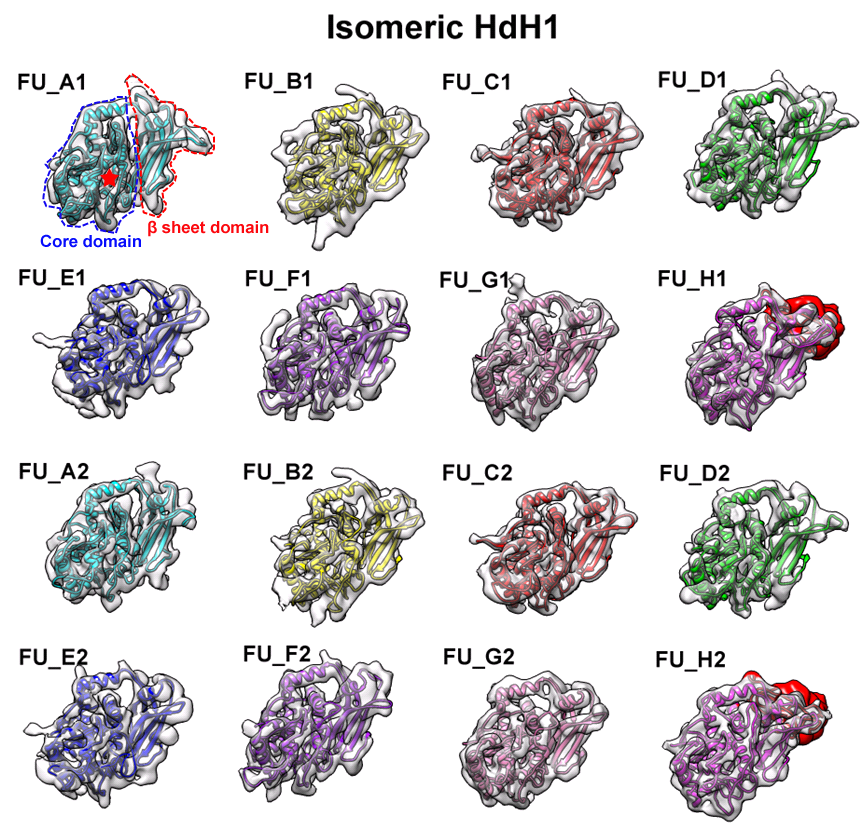

Supplement: Figure S3 — Pseudoatomic models of FUs of isomeric HdH1 within an asymmetric unit. Each FU consists of two domains: the core domain (red dotted line) and the β sheet domain (blue dotted line). The location of the PO active center is indicated by a red star, as shown in FU_A1. The cupredoxin-like domain in FU-H is highlighted in red. (TIF) [file pone.0098766.s003.tif]

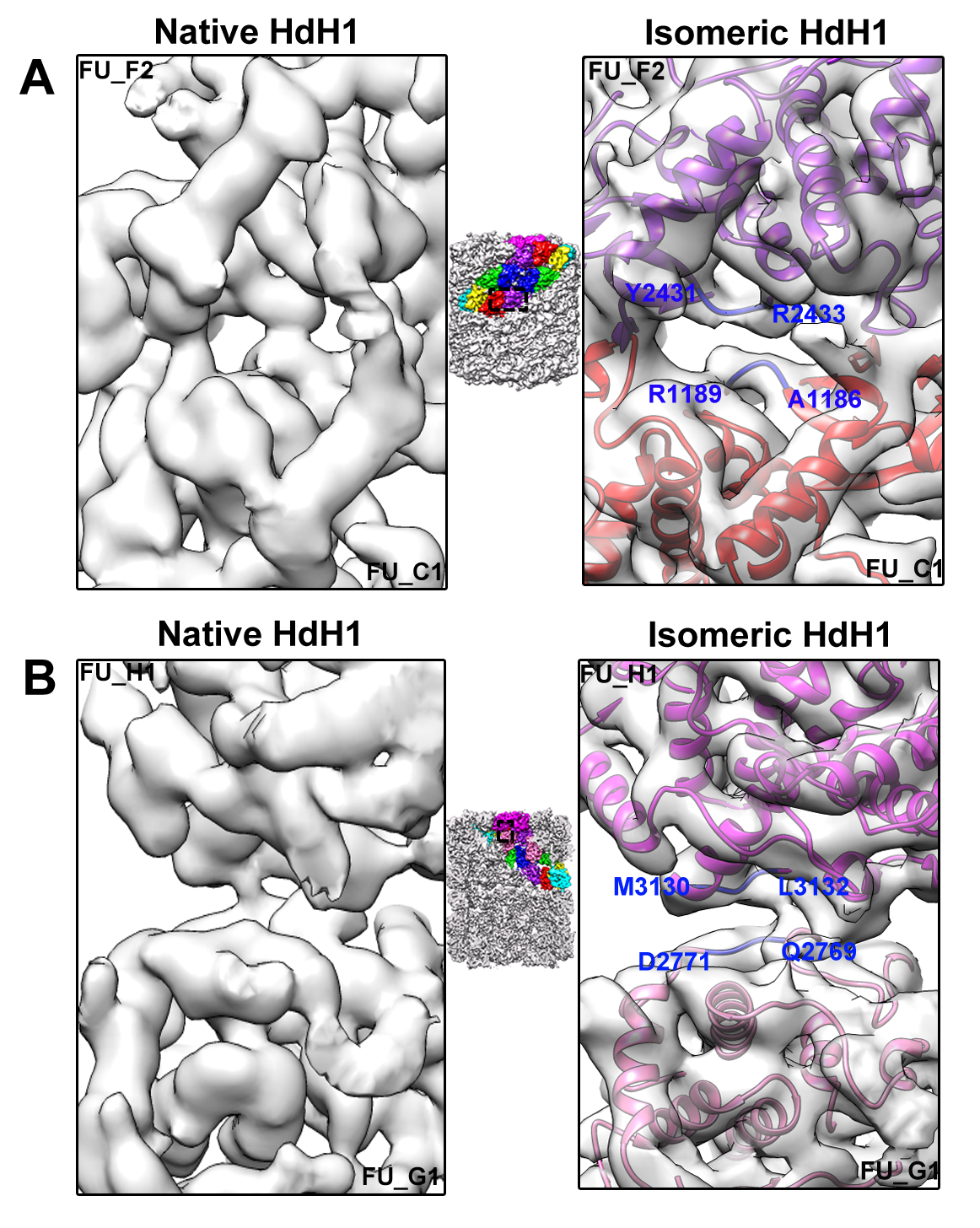

Supplement: Figure S4 — Decrease in FU-FU interaction inside one asymmetric unit of HdH1. Left panels: density maps of native HdH1 filtered to 6.8 Å from [23]; Right panels: density maps of isomeric HdH1 filled with the corresponding pseudoatomic models from Fig. S3. The locations of the interactions in the didecamer are indicated by black squares in the inserts. Residues possibly involved in the interactions are labeled in the right panels. (A) Decrease in the interaction between FU_F2 and FU_C1. (B) Decrease in the interaction between FU_G1 and FU_H1. (TIF) [file pone.0098766.s004.tif]

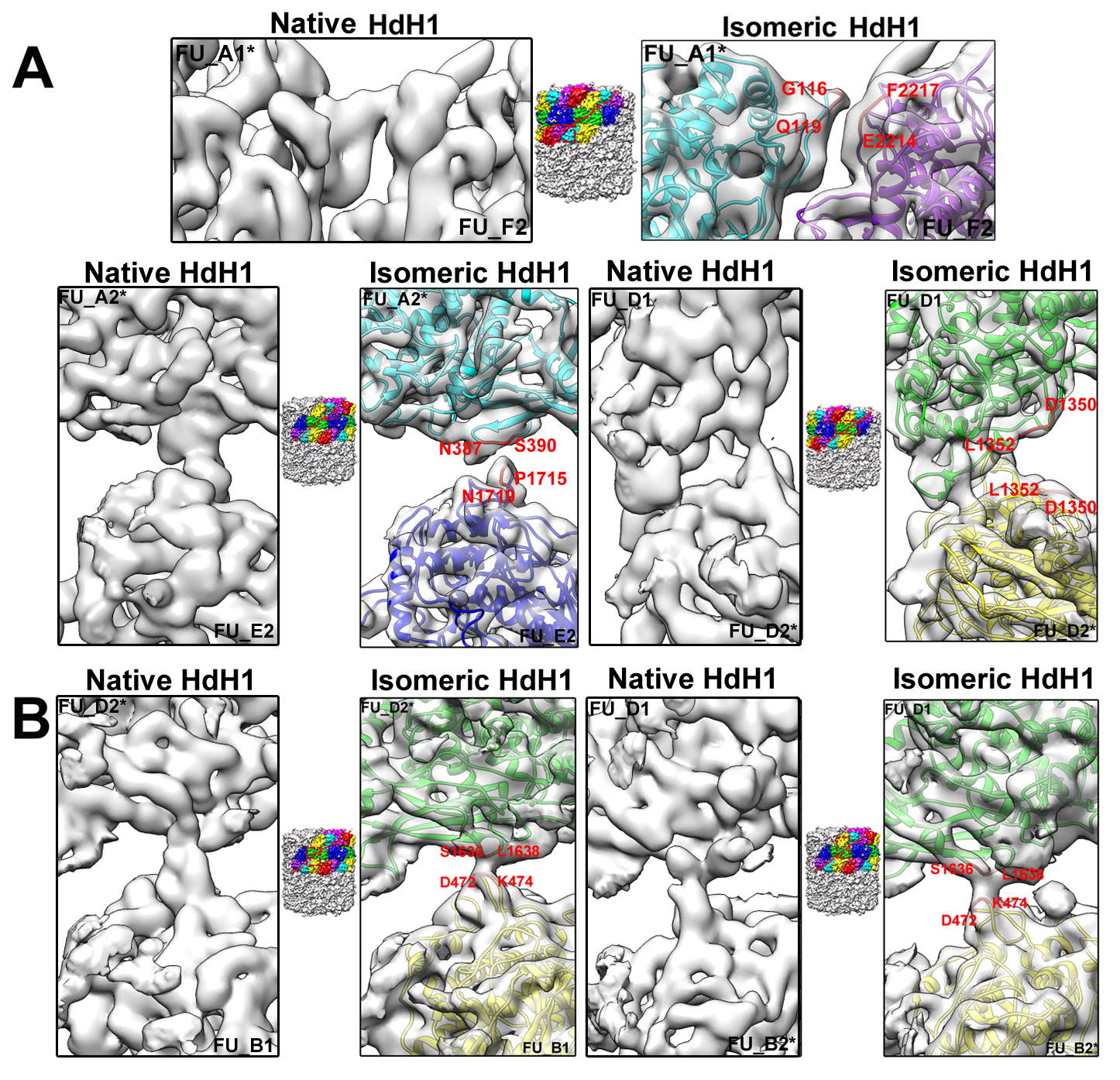

Supplement: Figure S5 — Decrease in FU-FU interaction between asymmetric units of HdH1. Left panels: density maps of normal HdH1 filtered to 6.8 Å from [23]; Right panels: density maps of isomeric HdH1 filled with the corresponding pseudoatomic models from Fig. S3. A red dot line is used to illustrate the boundary of two asymmetric units. The locations of the interactions are indicated by red squares in the inserts. The residues possibly involved in the interactions are labeled in the right panels. (A) Loss of FU-FU interaction between asymmetric units, i.e., FU_A1* and FU_F2, FU_A2* and FU_E2, and FU_D1 and FU_D2*, where * stands for FU from another asymmetric unit. (B) Two FU-FU interactions involved in the oxygen-binding “communication cluster” [1], i.e. FU_D2* and FU_B1, FU_D1 and FU_B2*, are retained in the isomeric HdH1 structure. (TIF) [file pone.0098766.s005.tif]

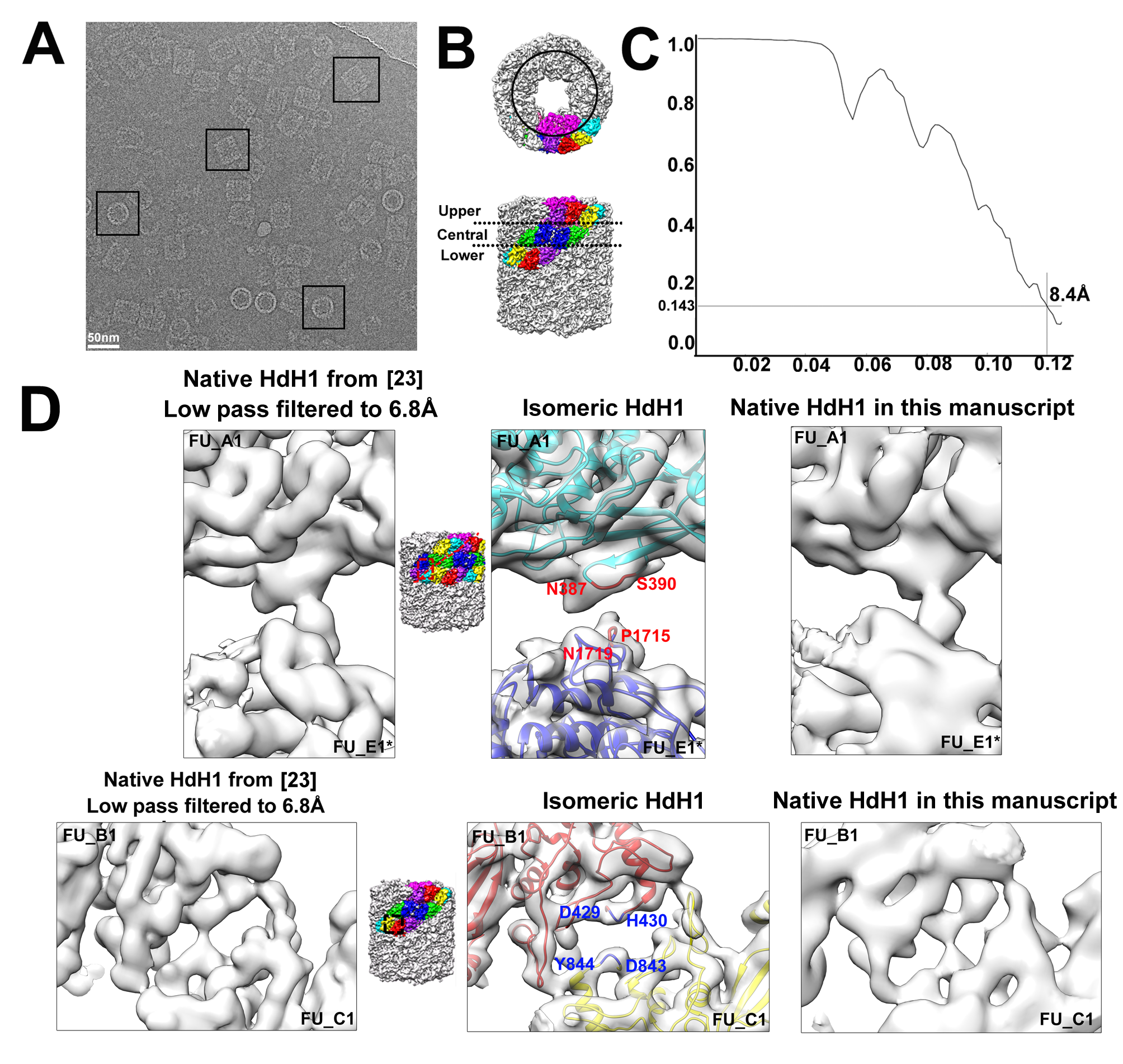

Supplement: Figure S6 — The overall structure of native HdH1 isolated from healthy abalones. (A) A representative cryo-EM micrograph of native HdH1. The didecameric particles used in the 3D reconstruction are marked with black boxes. (B) Top view and side view of 3D reconstructed map of native HdH1. An asymmetric unit is highlighted and its component Function Units (FUs) are marked with different colors. The slab area containing FU_H1 and FU_H2 is circled by a black line in the top view panel. The top, middle and bottom tiers are labeled. (C) FSC curve of native HdH1 reconstruction according to “gold-standard” criterion. (D) Comparison of cryo-EM density map of isomeric HdH1 isolated from AbSV-infected abalone (middle) with that of native HdH1 reconstructed by Zhang et al [23] (left, low pass filtered to 6.8 Å) and that of native HdH1 isolated from healthy abalone (right). Upper panel: An example of previously indicated FU-FU interaction loss between FUs from neighboring asymmetric units, i.e., FU_A1 (cyan) and FU_E1* (blue), in isomeric HdH1 (middle) as compared with the native HdH1 by Zhang et al [23] was found almost intact in the native HdH1 isolated from healthy abalone (right). A dot red line is used to indicate the boundary of two asymmetric units. The location of the interaction is highlighted by a red box in the small insert panel. Residues possibly involved in the interaction are labeled. Lower panel: An example of FU-FU loss of interaction between FUs inside one asymmetric unit, i.e., FU_B1 (red) and FU_C1 (yellow), is similarly illustrated. The FU-FU interaction lost in the isomeric HdH1 (middle) was found mostly intact in the native HdH1 from healthy abalone. The location of this FU-FU interaction in the didecamers is highlighted by a black box in the insert small panel. Residues possibly involved in the interaction are labeled. (TIF) [file pone.0098766.s006.tif]

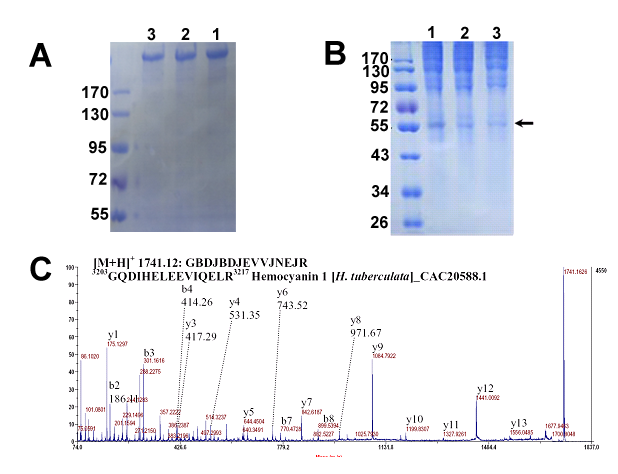

Supplement: Figure S7 — SDS-PAGE and mass spectrometry (MS) analysis of Hcs. (A) SDS-PAGE analysis of native Hcs. The lanes 1–3 were loaded with the eluted fractions 8.0, 8.2 and 8.4 of native Hcs in Fig. 2B (red) respectively. (B) SDS-PAGE analysis of isomeric Hcs. The lanes 1–3 were loaded with the eluted fractions 8.0, 8.3 and 8.6 of isomeric Hcs in Fig. 2B (blue) respectively. A protein band around 60 kD was indicated with a black arrow. (C) MS analysis result of the indicated 60 kD protein band in B, which corresponds to FU_H of HdH1. (TIF) [file pone.0098766.s007.tif]
